# Draft Genome Sequences of 12 Feline *Bartonella henselae* Isolates

**DOI:** 10.1128/genomeA.00075-17

**Published:** 2017-03-30

**Authors:** Cédric Woudstra, Patrick Fach, Bruno B. Chomel, Nadia Haddad, Henri-Jean Boulouis

**Affiliations:** a Food Safety Laboratory, Platform IdentyPath, Université Paris-Est, Anses, Maisons-Alfort, France; b Department of Population Health and Reproduction, School of Veterinary Medicine, University of California, Davis, California, USA

## Abstract

*Bartonella henselae* is the main causative agent of cat scratch disease. In this report, we present the draft genome sequences of 12 strains of *Bartonella henselae* originating from the United States, Denmark, and France. These strains were isolated from cats and belonged to either 16S rRNA genotype I or 16S rRNA genotype II.

## GENOME ANNOUNCEMENT

*Bartonella henselae* is a Gram-negative facultative intracellular bacterium of veterinary and zoonotic importance distributed worldwide. Domestic cats and other felids are the major reservoir hosts for this pathogen (1). The transmission of *B. henselae* from cat to cat is ensured by the cat flea *Ctenocephalides felis*. Ticks and biting flies have also been reported to be potential vectors (2). In humans, *B. henselae* is the main causative agent of cat scratch disease (CSD). This syndrome is characterized by a self-limiting regional lymphadenopathy in immunocompetent patients; however, infected immunocompromised individuals can develop severe multiple clinical manifestations, including bacillary angiomatosis, hepatic peliosis, hepatitis, endocarditis, fever, and bacteremia (3). Three distinct genotypes have been defined on the basis of their 16S rRNA sequence. Genotype I has been associated with more severe clinical manifestations than those induced by genotype II in humans. The third genotype (I/II strains with both type I and type II 16S rRNA) is the least common (4, 5). In order to more thoroughly investigate the genetic relationships between the different genotypes, we sequenced 12 feline strains from different geographical origins. These strains originated from the United States (U4 and F1), Denmark (A233, A235, A242, and A244), and France (A20, A71, A74, A76, A112, and A121). All of the strains were isolated from cats and belonged to either 16S rRNA genotype I or 16S rRNA genotype II.

Genomic DNA was extracted from one colony isolated on a blood agar plate by using the DNeasy blood and tissue kit (Qiagen), with an additional RNaseA (Roche) treatment. Libraries were prepared using the Nextera XT kit (Illumina). Whole-genome sequencing was performed using an Illumina MiSeq platform (Illumina), according to the manufacturer’s instructions. One MiSeq paired-end 300-nucleotide (nt) read MiSeq V3 chemistry run was carried out. The raw reads were trimmed (minimum length 35 bp, quality score 0.03) and assembled in CLC Genomics Workbench 7.5.1 by *de novo* assembly (minimum contig length 1000 bp), producing 41 to 69 contigs (Table 1). The median read depth of the assemblies ranged from 83× for isolate 71 to 216× for isolate 76 with *N*_*50*_ values between 82 kbp and 117 kbp (Table 1). The sequences were annotated with the National Center for Biotechnology Information (NCBI) Prokaryotic Genomes Automatic Annotation Pipeline (PGAP) at https://www.ncbi.nlm.nih.gov/genome/annotation_prok/.

**TABLE 1:** NCBI accession numbers and assembly metrics of *Bartonella henselae* draft genomes

Strain	No. of contigs	Genome size (Mbp)	*N*_50_ (bp)	Median read depth	No. of coding sequences	NCBI accession no.
F1	69	1.86	90	177	1,479	LOAI00000000
A20	60	1.84	111	88	1,454	LNZX00000000
A71	65	1.84	90	83	1,463	LOAA00000000
A74	63	1.85	117	126	1,461	LNZY00000000
A76	55	1.86	113	216	1,466	LNZZ00000000
112	62	1.84	84	165	1,467	LOAB00000000
U4	55	1.81	110	150	1,420	LOAH00000000
A121	52	1.84	99	173	1,458	LOAC00000000
A233	44	1.83	112	212	1,461	LOAD00000000
A235	41	1.82	102	118	1,448	LOAE00000000
A242	42	1.86	108	154	1,486	LOAF00000000
A244	41	1.85	82	161	1,470	LOAG00000000

The average size of the genomes in this study was 1.84 Mb, with 1.81 Mb being the smallest genome size (isolate U4, Table 1). On average, 1,460 coding sequences were identified in the genomes (Table 1).

A detailed report on further analyses of the draft genome sequences will be released in a future publication.

### Accession number(s).

The annotated draft whole-genome sequences of these *Bartonella henselae* strains were deposited in DDBJ/ENA/GenBank (Table 1). The versions described in this paper are the first versions.
